# LncRNA STXBP5-AS1 suppresses stem cell-like properties of pancreatic cancer by epigenetically inhibiting neighboring androglobin gene expression

**DOI:** 10.1186/s13148-020-00961-y

**Published:** 2020-11-07

**Authors:** Shi Chen, Long Huang, Ge Li, Funan Qiu, Yaodong Wang, Can Yang, Jingjing Pan, Zhangwei Wu, Jiangzhi Chen, Yifeng Tian

**Affiliations:** 1 Department of Hepato-Biliary-Pancreatic Surgery, Fujian Provincial Hospital, Shengli Clinical Medical College of Fujian Medical University, Fujian Medical University, No. 134 East Street, Fuzhou, 350001 Fujian China; 2 Department of Hepatobiliary Surgery, Union Hospital, Fujian Medical University, Fuzhou, 350001 Fujian China

**Keywords:** Long non-coding RNA, STXBP5-AS1, ADGB, Pancreatic cancer

## Abstract

Previous studies suggest the tumor suppressor role of long non-coding RNA (lncRNA) *STXBP5-AS1* in cervical and gastric cancer, but its expression pattern and functional mechanism are still elusive in pancreatic cancer (PC). Relative expression of *STXBP5-AS1* in PC both in vivo and in vitro was analyzed by real-time PCR. IC_50_ of Gemcitabine was determined by the MTT assay. Cell proliferation in response to drug treatment was investigated by colony formation assay. Cell apoptosis was measured by both caspase-3 activity and Annexin V/PI staining. Cell invasion capacity was scored by the transwell assay in vitro, and lung metastasis was examined with the tail vein injection assay. Cell stemness was determined in vitro by sphere formation and marker profiling, respectively, and in vivo by limited dilution of xenograft tumor incidence. Subcellular localization of *STXBP5-AS1* was analyzed with fractionation PCR. Association between *STXBP5-AS1* and *EZH2* was investigated by RNA-immunoprecipitation. The binding of EZH2 on *ADGB* promoter was analyzed by chromatin immunoprecipitation. The methylation was quantified by bisulfite sequencing. We showed downregulation of *STXBP5-AS1* in PC associated with poor prognosis. Ectopic *STXBP5-AS1* inhibited chemoresistance and metastasis of PC cells. In addition, *STXBP5-AS1* compromised stemness of PC cells. Mechanistically, *STXBP5-AS1* potently recruited *EZH2* and epigenetically regulated neighboring *ADGB* transcription, which predominantly mediated the inhibitory effects of *STXBP5-AS1* on stem cell-like properties of PC cells. Our study highlights the importance of the *STXBP5*-*EZH2*-*ADGB* axis in chemoresistance and stem cell-like properties of PC.

## Background

Pancreatic cancer (PC) is one of the most lethal human malignancies with poor prognosis [1]. There are 227,000 deaths claimed by this disease every year globally. Currently, surgical removal and chemotherapy are still the mainstay of clinical management options [2]. However, due to the lack of evident symptom and reliable early diagnosis, PC is frequently diagnosed at late and untreatable stage, which greatly contributes to the relatively unfavorable prognosis [3, 4]. On the other hand, assembled studies suggest the existence of specific subpopulation in pancreatic tumor cells with characteristic features of self-renewal, differentiation and capability of driving tumor incidence and metastasis [5–7]. More importantly, the stem cell-like tumor cells are deemed as the major cause of resistance to both conventional chemotherapy and radiotherapy [8–10]. Therefore, insightful understanding into pathogenesis of PC and characterization of tumor stem cells involved in this disease is extremely critical in the search for early diagnostic marker and therapeutic targets.

Long non-coding RNA (lncRNA) is a class of RNA molecules with an average length of more than 200 nt and no protein coding potential [11]. Increasing evidences support the fundamental roles of lncRNA in multiple aspects of tumor biology in almost all human cancer types, including cell differentiation, proliferation, apoptosis, metastasis and cell stemness [12–15]. More recent investigations suggest the association between lncRNAs and *EZH2*, which lead to assembly of polycomb repressive complex 2 (PRC2) and enhance local histone H3 lysine 27 methylation, and consequently inhibited target gene expression epigenetically [16–18]. Here, we focused on a novel lncRNA, *STXBP5-AS1*, in PC, which was previously investigated in cervical and gastric cancers. Huang et al. first reported that *STXBP5-AS1* suppressed cell proliferation, invasion and migration through blockading the PI3K/AKT pathway, which was predominantly mediated by negative regulation on STXBP5 expression in non-small-cell lung carcinoma [19]. Subsequently, Cen et al. confirmed the involvement of STXBP5-AS1/PI3K/AKT in tumor suppressive effects in gastric cancer [20]. While in cervical cancer, Shao et al. suggested that *STXBP5-AS1* functioned as a competing endogenous RNA to upregulate *PTEN* via sponging miR-96-5p to reduce cervical cancer cell proliferation and invasion [21]. Notably, Ham et al. found that both ginsenoside Rg3 and Korean red ginseng extracts were capable of epigenetically regulating the expression of tumor-related *RFX3-AS1* and *STXBP5-AS1* [22], therefore providing experimental evidences in support of the targetability of *STXBP5-AS1* by traditional Chinese medicine. Our study evidenced the anti-tumoral properties of *STXBP5-AS1* in PC, suppression of which rendered drug resistance and stem cell-like features to PC cells. We further demonstrated the epigenetic regulation of *ADGB* by *STXBP5-AS1* via interacting with and potently recruiting *EZH2*. Therefore, our data highlighted the critical contributions of *STXBP5-AS1* in PC.

## Materials and methods

### Clinical samples

A total of 60 PC tumors with paired adjacent normal tissues were collected from Fujian Provincial Hospital, Shengli Clinical Medical College of Fujian Medical University, Fujian Medical University. Written consents were obtained from all enrolled patients, and approval from the Institutional Ethics Committee was received before initiation of this study. Diagnosis was confirmed by independent pathologists, and the specimens were immediately flash-frozen in liquid nitrogen.

### Cell culture and treatment

PC cell lines (AsPC-1, SW1990, Capan-2, CFPAC-1, PANC-1 and Mia PaCa-2) and the normal human pancreatic ductal cell line hTERT-HPNE were ordered from the American Type Culture Collection (ATCC, VA, USA). All cancer cells were maintained in RPMI-1640 (Sigma, MO, USA) containing 10% fetal bovine serum (Invitrogen, CA, USA) and 1% antibiotics (penicillin–streptomycin, Hyclone, MA, USA). The hTERT-HPNE cells were cultured following the ATCC recommendation in 75% glucose-free DMEM (supplemented with L-glutamine and sodium bicarbonate, Sigma, MO, USA) and 25% M3 Base Medium (Incell, CA, USA). 5-Aza-CdR was purchased from Sigma (St. Louis, MO, USA), and cells were treated with the optimal concentration of 5-Aza-CdR (1 µM) for 72 h. Regular cell culture was performed in humidified CO_2_ (5%) incubator at 37 °C.

### Gene overexpression and knockdown

*STXBP5-AS1* and *ADGB* overexpression cell lines were established by infecting cells with lentivirus containing the *STXBP5-AS1* (pSIN-STXBP5-AS1) and *ADGB* sequences (pSIN-ADGB), followed by puromycin selection to acquire stable overexpression cells. *STXBP5-AS1* and *EZH2* knockdown was achieved by transfecting siRNAs of the following sequences using Lipofectamine 2000 (Thermo Fisher Scientific) according to the manufacturer’s instructions:

si-STXBP5-AS1-1: GCAAGTTGCTGAGTATTAT.

si-STXBP5-AS1-2: GGATCTTATTCTCCCACAT.

si-EZH2-1: GGTGAATGCCCTTGGTCAATA.

si-EZH2-2: GAAGCAAATTCTCGGTGTCAA.

### Real-time PCR

RNA was extracted with the TRIzol Reagent (Invitrogen, MA, USA) in accordance with the manufacturer’s manual. cDNA synthesis was conducted with 1 μg of RNA with cDNA Synthesis Kit (Takara, Ohtsu, Japan). Relative mRNA was quantified with SYBR Premix Ex Taq (TaKaRa) on Applied Biosystems 7900 PCR System (Applied Biosystems, CA, USA). The quantification of gene level was calculated by the 2^−ΔΔCT^ method, using GAPDH as the internal reference gene. The primer sequences were listed as below:

STXBP5-AS1 F: 5′-AGGGACTTGCCTTGTCGCTGAT-3′;

STXBP5-AS1 R: 5′-GAGATTTAGGTGGGGACGCTGC-3′;

GAPDH F: 5′-ACGGATTTGGTCGTATTGGGCG-3′;

GAPDH R: 5′-GCTCCTGGAAGATGGTGATGGG-3′;

Sox2 F: 5′-TGCACCGCTACGACGTGAGC-3′;

Sox2 R: 5′-GCCCTGGAGTGGGAGGAAGA-3′;

Bmi1 F: 5′-GCTTCAAGATGGCCGCTTG-3′;

Bmi1 R: 5′-TTCTCGTTGTTCGATGCATTTC-3′;

Lin28 F: 5′-AAAGGAGACAGGTGCTAC-3′;

Lin28 R: 5′-ATATGGCTGATGCTCTGG-3′;

Nanog F: 5′-AGTTGGACAGGGAGATGGC-3′;

Nanog R: 5′-AACCTTCCTTGCTTCCACG-3′;

ADGB F: 5′-AGACCCTCATCAGAAGTGCAG-3′;

ADGB R: 5′-GCTACCAGAGGACAAGACCTACT-3′.

#### Sphere formation assay

400 cells were seeded into 6-well plate, and 50 cells were seeded into 24-well plate, followed by continuous culture for 10 days. Spheres were maintained in serum-free DMEM/F12 medium containing 2% B27 (Invitrogen, MA, USA) plus EGF (20 ng/ml), bFGF (20 ng/ml) and insulin (5 μg/ml from PeproTech, NJ, USA).

### Cell viability and apoptosis

The indicated cells were prepared in 96-well plate (10^4^ cells/well) and treated with serial concentrations of Gemcitabine. After 48 h, cell viability was monitored by the MTT assay, and IC_50_ value of Gemcitabine was determined with SPSS 23. To measure cell apoptosis, the indicated cells were treated with 100 ng/ml of Gemcitabine. After 48 h, single-cell suspension was prepared and stained with Annexin V-FITC-PI Apoptosis Detection Kit (Sigma, MO, USA) as suggested by the provider, and followed by FACS analysis on CytoFlex (Beckman Coulter, CA, USA).

### Colony formation assay

Well-dispersed single cells were seeded into 6-well plate (500 cells/well) and subjected to drug treatment for 48 h at 37℃. Fresh medium was then replaced, followed by consecutive culture for another 10 days. Colonies were fixed with 3% formaldehyde briefly and stained with 0.5% crystal violet for 15 min (Sigma, MO, USA).

### Transwell assay

Invasion capacity was assessed using the transwell chamber which was pre-coated with 1% Matrigel (BD Biosciences, CA, USA). Cells (10^3^/well) were seeded into insert and cultured in serum-free medium. The lower compartment was supplied with complete medium as chemo-attractant. After 12 h, the non-invaded cells were washed off and invaded cells were fixed with cold-methanol and stained with 0.25% crystal violet.

### Lung colonization model

PANC-1 cells (either vector control or *STXBP5-AS1*-overexpressing) were prepared into single-cell suspension in PBS (1 × 10^6^ cells/ml), and i.v. injected into the lateral tail vein. After 21 days, all subject mice were sacrificed and lung macro-metastasis was examined with H&E staining. The animal study was approved by the Institutional Animal Care and Use Committee and in strict accordance with the NIH guideline.

#### Western blot

Cells were lysed in RIPA buffer on ice, and protein concentration was quantified by the BCA method (Sigma, MO, USA). 20 μg protein was resolved by SDS-PAGE and transferred onto PVDF membrane (Millipore, MA, USA). After brief blocking with 5% milk, the membrane was probed with primary antibodies: rabbit anti-Sox2 (#2748, Cell Signaling Technology, MA, USA), rabbit anti-Bmi1 (#6964, Cell Signaling Technology, MA, USA), rabbit anti-Lin28 (#3695, Cell Signaling Technology, MA, USA), rabbit anti-Nanog (#8822, Cell Signaling Technology, MA, USA), rabbit anti-ADGB (ab204085, Abcam, Cambridge, UK), rabbit anti-β-actin (#4970, Cell Signaling Technology, MA, USA) at 4 °C overnight. After washing, membranes were hybridized with secondary antibodies for another hour. The blots were detected with ECL Kit (APPLYGEN, Beijing, China) and visualized on LI-COR system (Biosciences, Lincoln, NE, USA).

#### Xenograft tumor model

To evaluate the tumorigenic capacity, PANC-1 cells (control or STXBP5-AS1-overexpression, 2 × 10^3^, 2 × 10^4^, 2 × 10^5^, 2 × 10^6^, 2 × 10^7^ cells) were subcutaneously injected into nude mice (*n* = 8 for each group). Tumor progression was continuously monitored for up to 2 weeks. All mice were then sacrificed, and xenograft tumor formation was validated by pathological examination.

#### Subcellular localization

PARIS Kit (Life Technologies, Carlsbad, CA, USA) was employed to fractionize cell nuclear and cytosol RNA. RNA was extracted and reversely transcribed as previously described. The relative distribution of *STXBP5-AS1* was measured by real-time PCR. *GAPDH* and *U6* were employed as reference for cytosol and nuclear localization, respectively.

#### RNA immunoprecipitation (RIP)

RIP assay was used to evaluate binding between *STXBP5-AS1* and *EZH2*. The assay was performed with the EZ-Magna RIP Kit (Millipore, MA, USA) following the manufacturer’s manual. Anti-EZH2 antibody and control IgG were obtained from Abcam. The immunoprecipitated RNA was recovered and further analyzed by qRT-PCR as previously described.

#### Chromatin immunoprecipitation (ChIP)

ChIP was conducted using EZ-Magna ChIP Chromatin Immunoprecipitation Kit (Millipore, MA, USA) according to the manufacturer’s recommendation. Chromatin cross-linked with 37% formaldehyde was ultrasonicated to generate DNA fragments with the average length of 500 to 1000 bp. The DNA species were precipitated with EZH2 antibody and recovered, which was further detected and quantified by qRT-PCR.

#### Bisulfite sequencing PCR (BSP)

DNA methylation of *ADGB* promoter was measured with commercially available kits. Genomic DNA from indicated cells was extracted with the DNeasy®Blood and Tissue Kit (Qiagen, CA, USA), which was followed by bisulfate modification. Bisulfite sequencing was then performed with EpiTect® Bisulite Kit (Qiagen, CA, USA) in accordance with the provider’s instructions.

#### Statistical analysis

Data are reported as mean ± standard deviation (SD). The inter-group comparison was analyzed using Student’s t test or one-way ANOVA analysis with a post hoc test using SPSS 23.0. *P* < 0.05 was regarded as statistically significant.

## Results

### Decreased *STXBP5-AS1* predicted poor prognosis in PC.

**Fig. 1:**
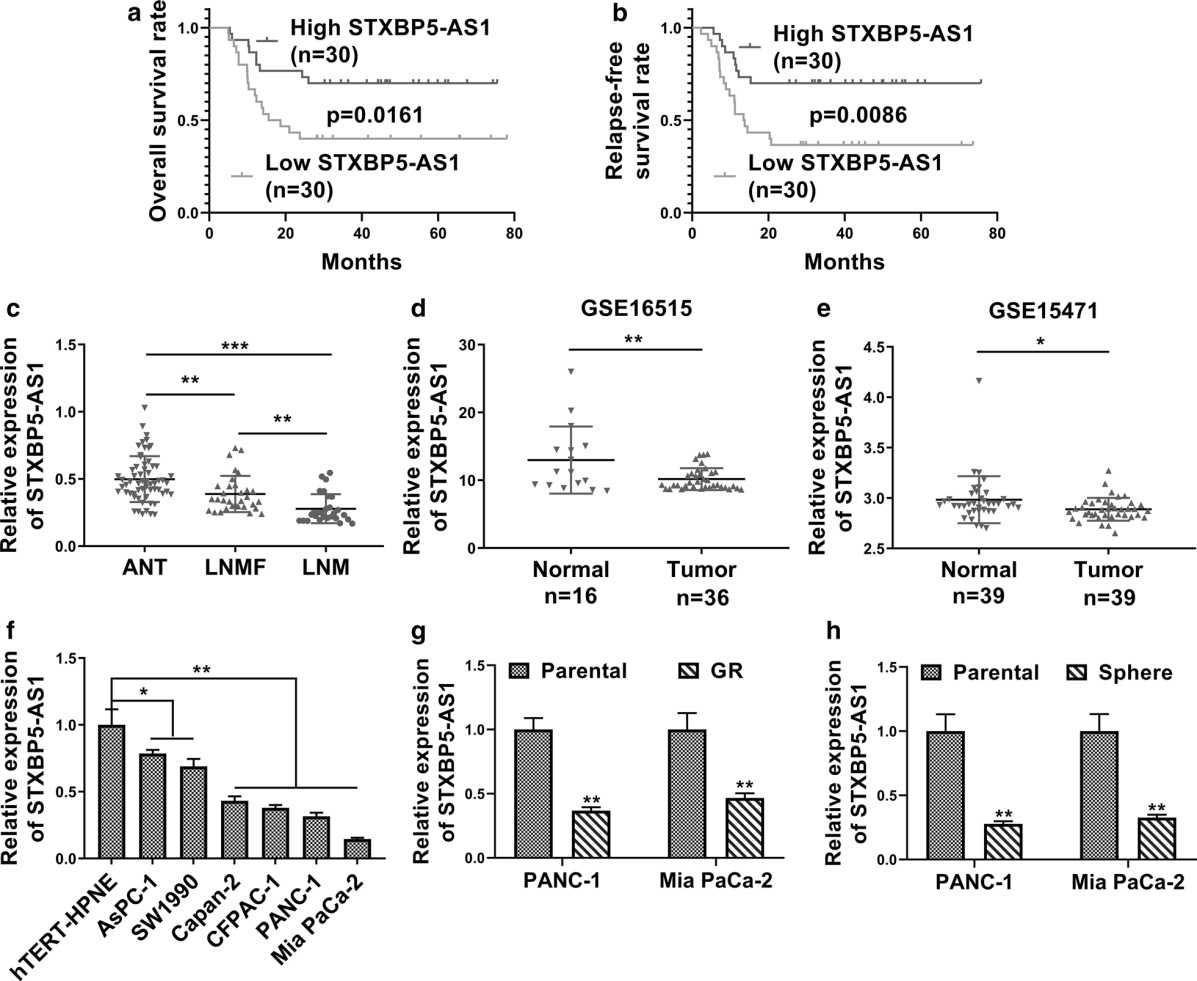
Decreased STXBP5-AS1 predicted poor prognosis in pancreatic cancer. **a** Kaplan–Meier’s analysis of the correlation between STXBP5-AS1 expression and the overall survival of PC patients. **b** Kaplan–Meier’s analysis of the correlation between STXBP5-AS1 expression and the relapse free survival of PC patients. **c** qRT-PCR analysis of STXBP5-AS1 expression in 60 pancreatic cancer tissues (PCT, 28 PC tissues from lymph node metastasis (LNM) patients and 32 PC tissues from lymph node metastasis-free (LNMF) patients) and respective adjacent normal tissues (ANT) (shown as 2^−ΔΔCT^). **d**, **e** STXBP5-AS1 expression in pancreatic tumor tissues and normal tissues from GSE16515 and GSE15471. **f** STXBP5-AS1 expression in six PC cell lines (AsPC-1, SW1990, Capan-2, CFPAC-1, PANC-1 and Mia PaCa-2) and a normal human pancreatic ductal cell line (hTERT-HPNE) was determined by qRT-PCR. **g** qRT-PCR analysis of STXBP5-AS1 expression in Gemcitabine-resistant (GR) or parental PANC-1 and Mia PaCa-2 cells. **h** The expression level of STXBP5-AS1 in spheres of PANC-1 and Mia PaCa-2 cells or parental PANC-1 and Mia PaCa-2 cells was measured by qRT-PCR.*P < 0.05; **P < 0.01

First of all, we analyzed a group of 60 PC patients, whose clinical–pathological features are listed in Additional file 1: Table S1. Analysis into these equally grouped patient samples, with respect to *STXBP5-AS1* transcript levels, manifested favorable both overall and relapse-free survival linking to relatively high *STXBP5-AS1* (Fig. 1a, b). Next, *STXBP5-AS1* expression in lymph node metastasis (LNM) patients and lymph node metastasis-free (LNMF) patients were significantly decreased compared to respective adjacent normal tissues (ANT) (Fig. 1c). Furthermore, data from GEO datasets showed significant decreased *STXBP5-AS1* in PC as compared to the benign tissues as well (Fig. 1d, GSE16515; Fig. 1e, GSE15471). We then determined the relative expression of *STXBP5-AS1* in a panel of PC cells and found significant downregulation in all tested cancer cell lines in comparison with normal pancreatic duct cell line Htert-HPNE (Fig. 1f). The suppressive expression of *STXBP5-AS1* was also noticed in Gemcitabine-resistant (GR) cells compared to parental ones in both PANC-1 and Mia PaCa-2 cell lines (Fig. 1g). The lower abundance of *STXBP5-AS1* was especially characterized in sphere derived from these two cells (Fig. 1h), which might imply a possible correlation between down-regulated *STXBP5-AS1* and cell stemness. Therefore, our data suggested that downregulation of *STXBP5-AS1* in PC might be mechanistically associated with GR and tumor cell stemness, as well as poorer clinical outcome.

### *STXBP5-AS1* inhibited chemoresistance and metastasis of PC cells.

**Fig. 2:**
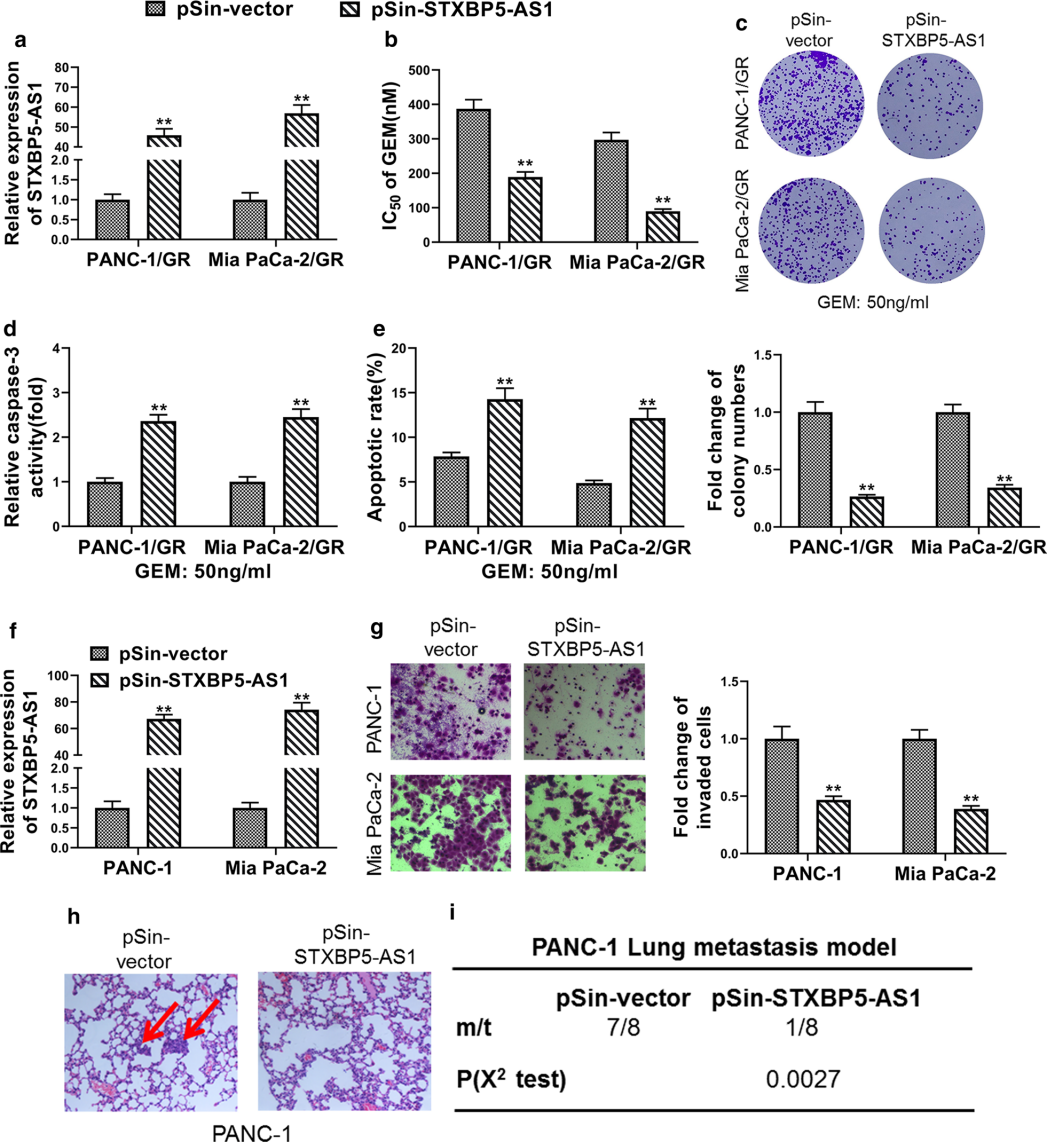
STXBP5-AS1 inhibited chemoresistance and metastasis of PC cells. **a** The overexpression efficiency of STXBP5-AS1 in Gemcitabine-resistant PANC-1 and Mia PaCa-2 (PANC-1/GR and Mia PaCa-2/GR) cells transfected with STXBP5-AS1 plasmid (pSin-STXBP5-AS1) or empty vector (pSin-vector) was confirmed by qRT-PCR. **b** Determination of the effects of STXBP5-AS1 overexpression on Gemcitabine IC_50_ in PANC-1/GR and Mia PaCa-2/GR cells using MTT assay. **c** Quantification of colony formation in Gemcitabine-challenged (50 ng/ml) PANC-1/GR and Mia PaCa-2/GR cells transfected with STXBP5-AS1 plasmid (pSin-STXBP5-AS1) or empty vector (pSin-vector). **d**, **e** PANC-1/GR and Mia PaCa-2/GR cells transfected with STXBP5-AS1 plasmid (pSin-STXBP5-AS1) or empty vector (pSin-vector) cells were treated with 50 ng/ml Gemcitabine for 48 h, the apoptosis was analyzed by caspase-3 activity assay and FACS. **f** The overexpression efficiency of STXBP5-AS1 in PANC-1 and Mia PaCa-2 cells stably transfected with STXBP5-AS1 plasmid (pSin-STXBP5-AS1) or empty vector (pSin-vector) was confirmed by qRT-PCR. **g** Transwell assay indicated that STXBP5-AS1 repressed the invasion of PANC-1 and Mia PaCa-2 cells. **h** and **i** H&E staining of the metastatic nodules in the lung of PANC-1 cells stably transfected with STXBP5-AS1 plasmid (pSin-STXBP5-AS1) or empty vector (pSin-vector) following tail vein injection into nude mice (200× scale bars) and incidence of lung metastasis in mice following tail vein injection of the respective PANC-1 cells. *P < 0.05; **P < 0.01 (*χ*^2^ test for *I*, Student’s *t* test for others)

Next, we complemented GR cell lines derived from both PANC-1 and Mia PaCa-2 cells with ectopic *STXBP5-AS1* (Fig. 2a). Drug resistance to Gemcitabine was significantly compromised by *STXBP5-AS1* as indicated by reduction of IC_50_ value of Gemcitabine (Fig. 2b). The colony formation capacity of PANC-1/GR and Mia PaCa-2/GR cells was greatly inhibited by ectopic *STXBP6-AS1* (Fig. 2c). Concurrently, activation of capase-3 in response to Gemcitabine exposure was tremendously augmented by over-expression of *STXBP5-AS1* in both GR cells (Fig. 2d). Correspondingly, remarkably increased cell apoptosis was observed *STXBP5-AS1*-proficient cells in comparison with parental ones upon treatment with 50 ng/ml Gemcitabine (Fig. 2e). In addition to GR cells, we generated *STXBP5-AS1*-overexpressing cells in naïve PANC-1 and Mia PaCa-2 cells as well (Fig. 2f). Cell invasion was evidently inhibited by ectopic *STXBP5-AS1* in both PANC-1 and Mia PaCa-2 cells (Fig. 2g). More importantly, we provided evidences in support of the metastasis-inhibiting effect of *STXBP5-AS1* in PANC-1 lung metastasis model. *STXBP5-AS1*-proficiency greatly suppressed lung metastatic loci establishment of tail vein-injected PANC-1 cells, as shown in the representative H&E staining of lung sections (Fig. 2h). Statistics suggested that lung metastasis occurred in 7 out 8 of vector control mice, while absent in only 1 out 8 *STXBP5-AS1*-complemented mice (Fig. 2i). Our data showed that *STXBP5-AS1* significantly improved chemosensitivity of GR cells, whereas it greatly blockaded metastasis of naïve PC cells.

### *STXBP5-AS1* suppressed stemness of PC cells

**Fig. 3:**
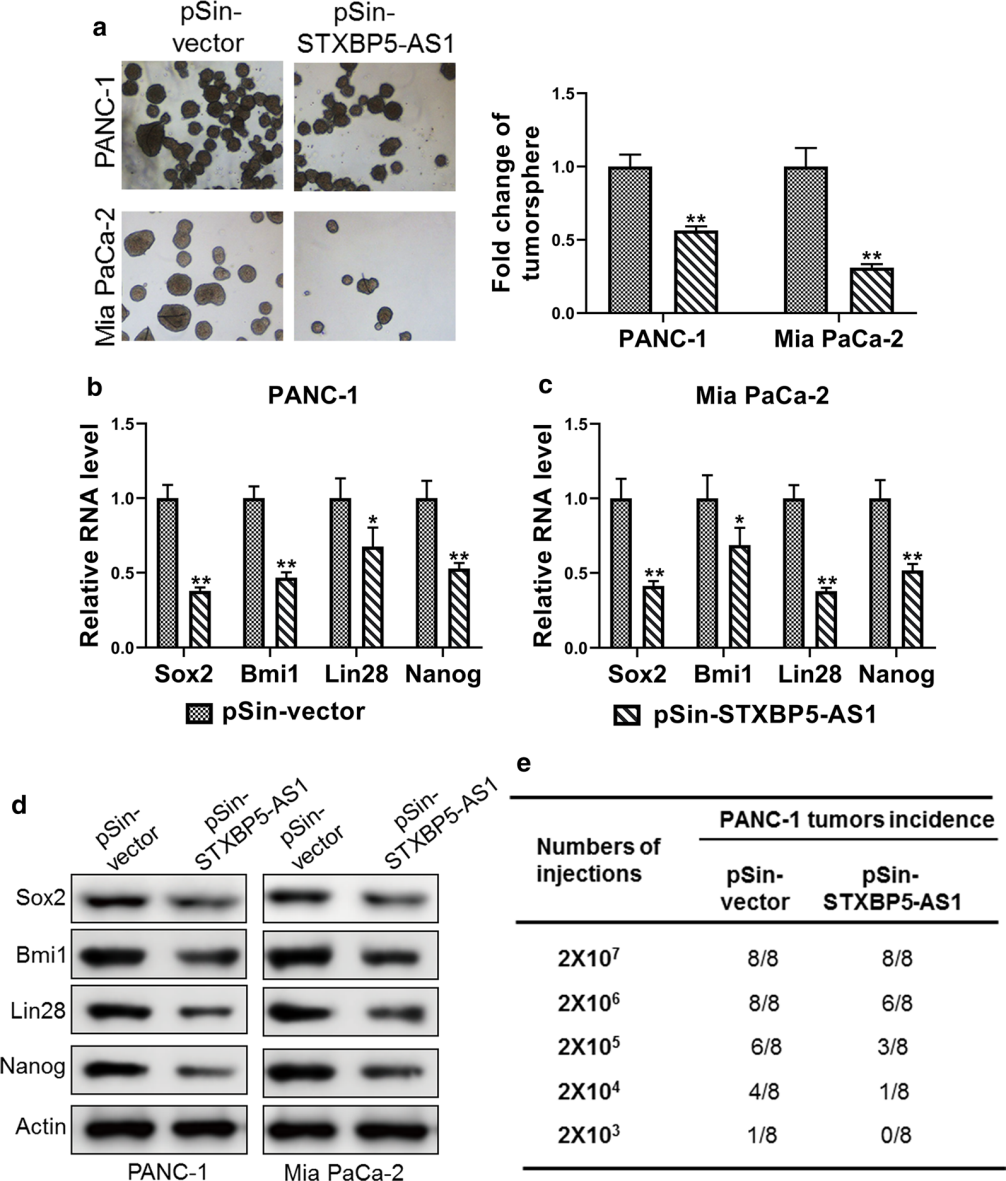
STXBP5-AS1 suppressed stemness of PC cells. **a** Sphere formation of PANC-1 and Mia PaCa-2 cells stably transfected with STXBP5-AS1 plasmid (pSin-STXBP5-AS1) or empty vector (pSin-vector). The total sphere numbers in each well were counted and images were taken at 40× magnification. **b**–**d** The expression levels of stem markers (Sox2, Bmi1, Lin28 and Nanog) in PANC-1 and Mia PaCa-2 cells stably transfected with STXBP5-AS1 plasmid (pSin-STXBP5-AS1) or empty vector (pSin-vector) were assessed by qRT-PCR and western blot. **e** Tumor incidence of PANC-1 cells stably transfected with STXBP5-AS1 plasmid (pSin-STXBP5-AS1) or empty vector (pSin-vector); cells were injected into the flank of mice with limiting dilutions as indicated. The number of tumors formed in each group was counted after 4 weeks. *P < 0.05; **P < 0.01

Our preliminary data showed downregulation of *STXBP5-AS1* in PC cell-derived spheres, which hinted the potential causal relation between *STXBP5-AS1* and tumor cell stemness. To further clarify this issue, we over-expressed *STXBP5-AS1* in both PANC-1 and Mia PaCa-2 cells, and examined the influence of *STXBP5-AS1* on sphere formation. As shown in Fig. 3a, the sphere formation capacity was greatly compromised by *STXBP5-AS1* in both cells. Molecular profiling of cell stemness markers including *Sox2*, *Bmi1*, *Lin28* and *Nanog* demonstrated remarkable reduction of all of four markers in response to *STXBP5-AS1* overexpression in both PANC-1 and Mia PaCa-2 cells at the transcriptional level (Fig. 3b, c). The suppressed expression of Sox2, Bmi1, Lin28 and Nanog was validated at protein level by Western blot analysis (Fig. 3d). Most importantly, compromised stemness by *STXBP5-AS1* was demonstrated by limiting dilution assay of xenograft tumor incidence. We noticed that *STXBP5-AS1*-proficiency greatly inhibited the incidence of PANC-1 cell-derived xenograft tumor, while total injected cell number was limited to 2 × 10^6^ and less (Fig. 3e). Taken together, our data supported the suppressive effects of *STXBP5-AS1* on stemness of PC cells both in vitro and in vivo.

### *STXBP5-AS1* epigenetically regulated neighboring gene *ADGB* transcription by binding to *EZH2*

**Fig. 4:**
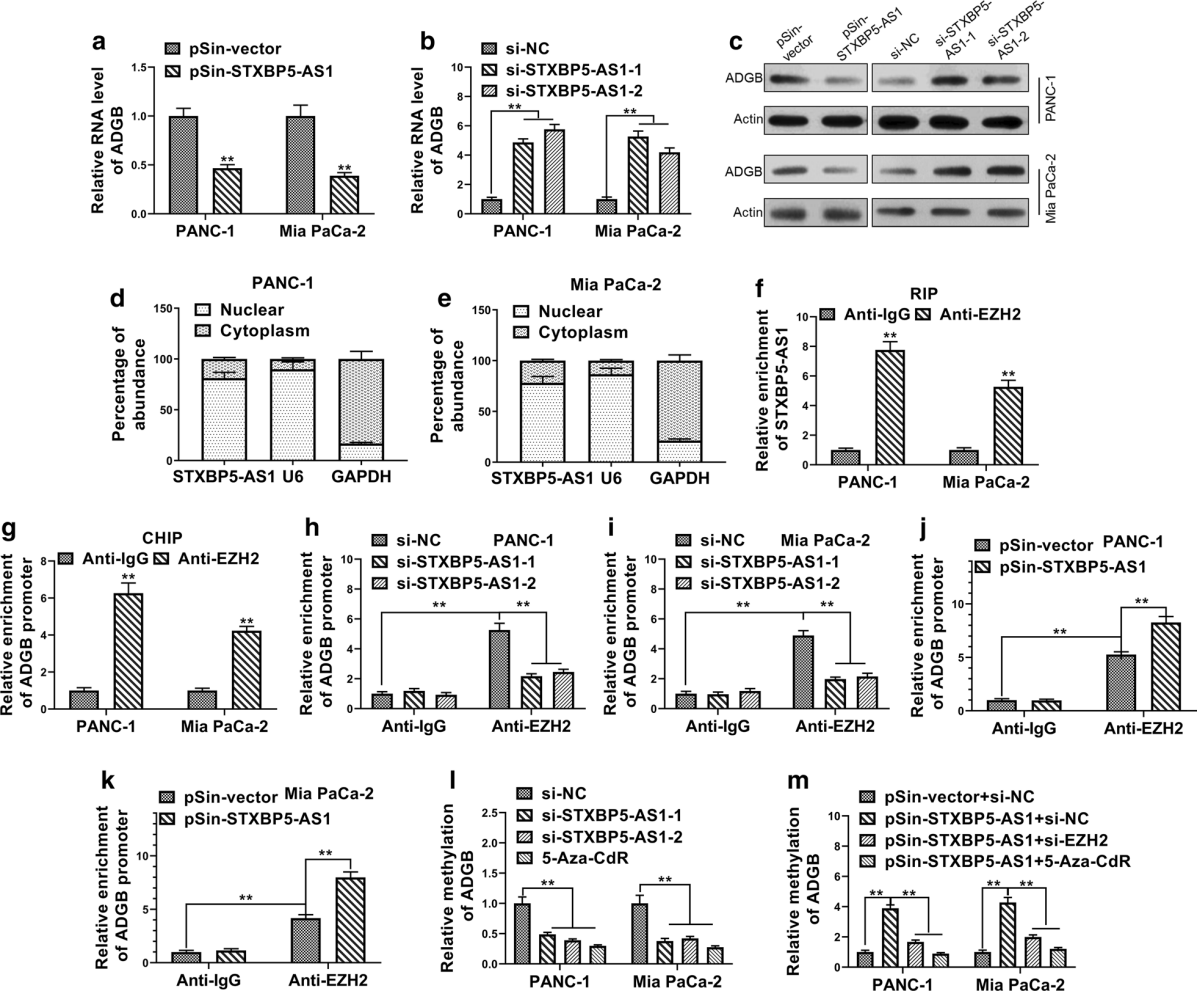
STXBP5-AS1 epigenetically regulated neighboring gene ADGB transcription by binding to EZH2. **a**–**c** The expression level of ADGB in STXBP5-AS1 overexpressing and downregulated -PANC-1 and Mia PaCa-2 cells was determined by qRT-PCR and western blot. **d**, **e** Distribution of STXBP5-AS1 in PANC-1 and Mia PaCa-2 cells was determined by qRT-PCR. **f** RIP assay was performed to determine the association between STXBP5-AS1 and EZH2. The fold enrichment of STXBP5-AS1 in PANC-1 and Mia PaCa-2 cells with antibodies against EZH2 was relative to non-specific IgG control. **g** ChIP-PCR assay was performed to determine the interaction between ADGB promoter and EZH2 with antibodies against EZH2 or IgG in PANC-1 and Mia PaCa-2 cells. **h**, **i** ChIP-PCR assay was performed to determine EZH2 enrichment at ADGB promoter region with antibodies against EZH2 or IgG in PANC-1 and Mia PaCa-2 cells transfected with STXBP5-AS1 siRNAs (si-STXBP5-AS1-1 and si-STXBP5-AS1-2) or negative control siRNA (si-NC). (J and K) ChIP-PCR assay was performed to determine EZH2 enrichment at ADGB promoter region with antibodies against EZH2 or IgG in PANC-1 and Mia PaCa-2 cells transfected with STXBP5-AS1 plasmid (pSin-STXBP5-AS1) or empty vector (pSin-vector). (L) The bisulfite sequencing PCR analysis (BSP) of the methylation levels of ADGB in 5-Aza-CdR-treated or STXBP5-AS1-downregulated PANC-1 and Mia PaCa-2 cells. (M) The bisulfite sequencing PCR analysis (BSP) of the methylation levels of ADGB in STXBP5-AS1-upregulated, STXBP5-AS1-upregulated plus EZH2 knockdowned or STXBP5-AS1-upregulated plus 5-Aza-CdR-treated PANC-1 and Mia PaCa-2 cells. The data represent the mean ± SD from three independent experiments. *P < 0.05; ** P < 0.01, Student’s *t* test

Next, we sought to understand the molecular mechanism underlying the tumor-suppressor role of *STXBP5-AS1* in PC. We focused on the neighboring genes in view of the well-recognized mode of action of lncRNA in regulating adjacent genes. We found *ADGB* was greatly inhibited by *STXBP5-AS1* in both PANC-1 and Mia PaCa-2 cells (Fig. 4a). In contrast, transcripts of *ADGB* were markedly up-regulated in *STXBP5-AS1*-depleted cells (Fig. 4b). The regulatory effects of *STXBP-AS1* on *ADGB* were further confirmed by Western blot analysis (Fig. 4c). To gain further insight into the regulatory mechanism, we then examined the subcellular localization of *STXBP5-AS1* transcripts via fractionation PCR analysis. As suggested by Fig. 4d, e, the majority of *STXBP5-AS1* from both PANC-1 and Mia PaCa-2 cells existed in the nuclear fraction with a minor proportion detectable in the cytoplasm, which indicated that *STXBP5-AS1* exerted physiological roles predominantly in the nucleus. Multiple lncRNAs have been previously identified to be involved in complex with *EZH2* and therefore in regulation of promoter methylation of target genes. Along this direction, we detected the enrichment of *STXBP5-AS1* transcripts in *EZH2* immunoprecipitated RNA species in both PANC-1 and Mia PaCa-2 cells (Fig. 4f). Meanwhile, direct association of EZH2 with *ADGB* promoter was demonstrated by ChIP assay as shown in Fig. 4g, which implicated the role of PRC2 complex in the epigenetic regulation of *ADGB*. The relative enrichment of *ADGB* promoter was significantly decreased by siRNA-mediated knockdown of *STXBP5-AS1* in comparison with scramble control (Fig. 4h, i). The association of EZH2 with *ADGB* promoter therefore was greatly dependent on *STXBP5-AS1*. Consistently, over-expression of *STXBP5-AS1* increased the enrichment of *ADGB* promoter in EZH2 immunoprecipitated complex, which suggested an enhancement of EZH2 binding to *ADGB* promoter (Fig. 4j, k). Consequently, methylation level of *ADGB* promoter region was tremendously decreased in response to *STXBP5-AS1* knockdown, which was comparable with treatment by the DNA demethylating agent 5-Aza-CdR (Fig. 4l). *STXBP5-AS1* overexpression oppositely increased methylation status of *ADGB* promoter, which was readily abrogated by simultaneous *EZH2*-knockdown or 5-Aza-CdR treatment (Fig. 4m). Summarily, we provided evidence that *STXBP5-AS1* potently inhibited expression of neighboring *ADGB* via an epigenetic mechanism, specifically through complexation with EZH2.

### *STXBP5-AS1* inhibited stem cell-like properties of PC cells by suppressing *ADGB* expression

**Fig. 5:**
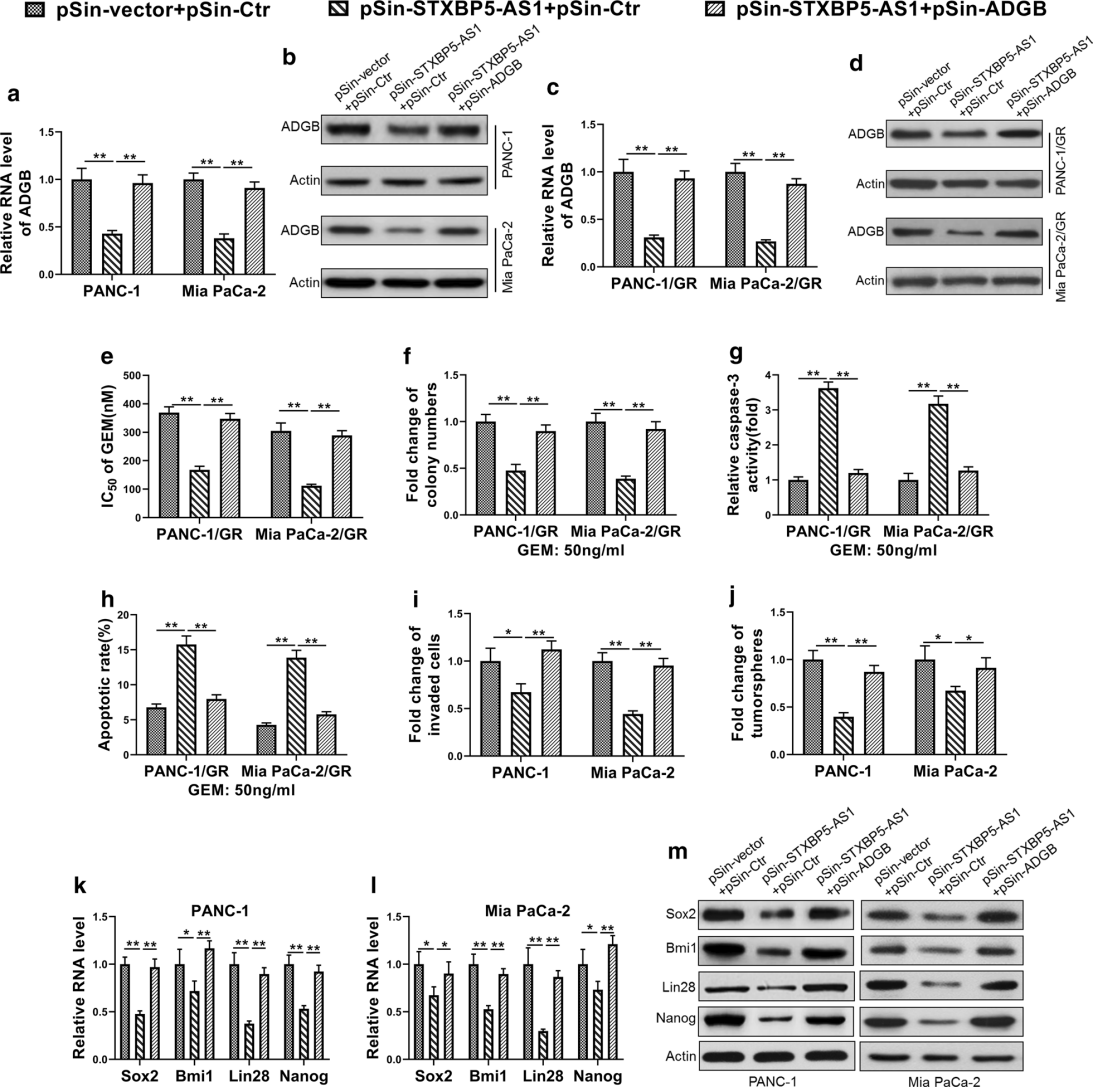
STXBP5-AS1 inhibited stem cell-like properties of PC cells by suppressing ADGB expression. **a**, **b** The expression level of ADGB in PANC-1 and Mia PaCa-2 cells co-transfected with two empty pSin vector (pSin-vector + pSin-Ctr), STXBP5-AS1 overexpression plasmid and an empty pSin vector (pSin-STXBP5-AS1 + pSin-Ctr) or STXBP5-AS1 overexpression plasmid and ADGB overexpression plasmid (pSin-STXBP5-AS1 + pSin-ADGB) was determined by qRT-PCR and western blot. **c**, **d** The expression level of ADGB in PANC-1/GR and Mia PaCa-2/GR cells co-transfected with two empty pSin vector (pSin-vector + pSin-Ctr), STXBP5-AS1 overexpression plasmid and an empty pSin vector (pSin-STXBP5-AS1 + pSin-Ctr) or STXBP5-AS1 overexpression plasmid and ADGB overexpression plasmid (pSin-STXBP5-AS1 + pSin-ADGB) was determined by qRT-PCR and western blot. **e** MTT assay showed that overexpressing ADGB rescued the decreased Gemcitabine IC_50_ in PANC-1/GR and Mia PaCa-2/GR cells due to forced expression of STXBP5-AS1. **f** Colony formation assay showed overexpressing ADGB rescued the decreased cell survival of Gemcitabine-challenged (50 ng/ml) PANC-1/GR and Mia PaCa-2/GR cells due to forced expression of STXBP5-AS1. **g**, **h** Caspase-3 activity assay and FACS indicated that overexpressing ADGB rescued the increased apoptosis of Gemcitabine-challenged (50 ng/ml) PANC-1/GR and Mia PaCa-2/GR cells due to forced expression of STXBP5-AS1. **i** Transwell assay showed overexpressing ADGB rescued the decreased cell invasion of PANC-1 and Mia PaCa-2 cells due to forced expression of STXBP5-AS1. **j** Tumor sphere formation assay showed overexpressing ADGB rescued the decreased spheres of PANC-1 and Mia PaCa-2 cells due to forced expression of STXBP5-AS1. **k**–**m** qRT-PCR and western blot indicated that overexpressing ADGB rescued the decreased expression levels of stem markers (Sox2, Bmi1, Lin28 and Nanog) in PANC-1 and Mia PaCa-2 cells due to forced expression of STXBP5-AS1. The data represent the mean ± SD from three independent experiments. *P < 0.05; ** P < 0.01, Student’s *t *test

Next, we sought to clarify whether down-regulated *ADGB* mainly mediated the inhibitory effects of *STXBP5-AS1* on cell stemness in PC cells. To this end, we established *STXBP5-AS1*-overexpressing and *ADGB*-overexpressing cells either individually or in combination in parental PANC-1 and Mia PaCa-2 cells (Fig. 5a, b), as well as in the respective GR cells (Fig. 5c, d). Consistent with previous observation, forced expression of *STXBP5-AS1* greatly improved the sensitivity of GR cells, which was almost completely reversed by co-expression of *ADGB* (Fig. 5e). Likewise, colony formation capacity was compromised in response to ectopic *STXBP5-AS1* in both PANC-1/GR and Mia PaCa-2/GR cells, while restored by complementation of *ADGB* (Fig. 5f). Caspase-3 activation and apoptotic index, which were greatly stimulated by introduction of *STXBP5-AS1*, were inhibited by *ADGB* overexpression as well (Fig. 5g, h). The invasive capacity compromised by *STXBP5-AS1* was greatly recovered by simultaneous expression of *ADGB* (Fig. 5i). Similarly, *ADGB* complementation evidently restored the colony formation in *STXBP5-AS1*-proficient cells (Fig. 5j). At the molecular level, co-overexpression of *ADGB* in the context of ectopic *STXBP5-AS1* expression up-regulated *Sox2, Bim1, Lin28* and *Nanog*, which was significantly inhibited by *STXBP5-AS1*-overexpression alone (Fig. 5k, l). This change was also validated at the protein level by Western blot analysis (Fig. 5m). Therefore, our results supported that *STXBP5-AS1* decreased the stem cell-like properties of PC cells mainly by epigenetic suppression of *ADGB*.

## Discussion

Despite previously reported tumor suppressor roles in cervical cancer, gastric cancer and non-small-cell lung cancer [19, 21], the relative expression pattern and functional mechanism of *STXBP5-AS1* in PC were still obscure currently. Here, we first characterized aberrant downregulation of *STXBP5-AS1* in PC both in vitro and in vivo. Particularly, a potential association was observed between *STXBP5-AS1* deficiency and cell stemness and drug resistance. In addition, high expression of *STXBP5-AS1* was significantly enriched in the PC patients without LNM, and consequently associated with both overall and relapse-free survival clinically. In cell culture, overexpression of *STXBP5-AS1* rendered GR cells sensitivity to Gemcitabine and greatly inhibited the colony formation capacity, which was accompanied with caspase-3 activation and cell apoptosis induction. In parental PC cells, the invasive behavior was suppressed by ectopic *STXBP5-AS1*. This phenotype was validated in vivo with tail vein injection of both empty control and *STXBP5-AS1*-overexpressing PANC-1 cells into nude mice as well. Notably, we observed the sphere formation efficacy was significantly compromised by *STXBP5-AS1* with concurrent downregulation of stemness markers including *Sox2, Bim1, Lin28* and *Nanog*. The suppressed stem cell-like properties were especially validated by the limiting dilution of xenograft tumor incidence. Mechanistically, we showed *STXBP5-AS1* epigenetically inhibited neighboring *ADGB* expression through potently recruiting EZH2 to the *ADGB* promoter and therefore enhancing DNA methylation. Complementation with *ADGB* remarkably restored drug resistance and colony formation in *STXBP5-AS1*-proficient GR cells, and simultaneously suppressed apoptotic activation. In parental PC cells, *ADGB* overexpression rescued the compromised cell invasion and decreased cell stemness markers. In summary, we provided experimental data supporting the tumor suppressor function of *STXBP5-AS1* in PC via inhibiting chemoresistance and stem cell-like properties, which was greatly mediated by epigenetic silencing of its neighboring *ADGB*.

Emerging evidences have suggested the involvement of lncRNAs in complexation with EZH2 and exerted epigenetic regulation in human cancers. Our data confirmed this notion via demonstrating the direct interaction between *STXBP5-AS1* and EZH2, which specified recruitment of EZH2 to the *ADGB* promoter and led to intensive DNA methylation and gene expression silencing. Our observations resembled multiple scenarios previously described. For instance, Liu et al. reported that *LINC01088* enhanced cell proliferation through scaffolding EZH2 and inhibiting p21 in human non-small-cell lung cancer [23]. Song et al. showed that *LINC01535* induced cervical cancer progression by specific targeting the miR-214/EZH2 feedback loop [24]. In colorectal cancer, Di et al. demonstrated that *SNHG14* facilitated distal metastasis via regulating EZH2-targeted *EPHA1* [25]. Xu et al. suggested *FOXD2-AS1* functioned as an oncogene in hepatocellular carcinoma by epigenetic suppression of *CDKN1B* (p27) with EZH2 [26]. Although we exhibited the direct association between *STXBP5-AS1* with EZH2, the detailed complexation was still to be elaborated by structural analysis.

ADGB (androglobin) was firstly described as a chimeric globin in metazoans and preferentially expressed in mammalian testis with poorly understood physiologic functions [27]. Until recently, the study performed by Huang et al. proposed that *ADGB* knockdown in glioma cell lines significantly inhibited cell proliferation and stimulated apoptosis [28], suggesting the potential oncogenic properties of *ADGB* in this disease. Consistent with this work, our data exhibited that overexpression of *ADGB* in *STXBP5-AS1*-proficient GR cells greatly stimulated drug resistance and inhibited cell apoptosis, while rendered stem cell-like properties and invasive potential to parental PC cells in the context of *STXBP5-AS1*-overexpression. Notably, the intensive methylation was detected in the promoter region of *ADGB* indicated the possible efficacy of DNA demethylating agents for therapeutic purpose, which is definitely worthy of further investigations in the near future. In view of the critical contributions of *ADGB* to drug resistance of PC, we also raised the hypothesis that combinational administration of both Gemcitabine and 5-Aza-CdR would greatly benefit the intrinsic resistant patients clinically.

Noteworthily, despite the significant downregulation of *STXBP5-AS1* characterized here in PC along with previous reports in both cervical and gastric cancers, the molecular mechanisms underlying this phenotype were still elusive currently. Another study limitation is that not all the clinical information was available in the public databases; therefore, the association of STXBP5-AS1 with survival cannot be analyzed. In the future, we would focus on this issue with the aid of bioinformatic analysis of publicly available tumor genome databases. In summary, we demonstrated that the *STXBP5-AS1*/EZH2/I axis in PC contributed to the chemoresistance and stem cell-like features.

## Supplementary information

**Additional file 1: Table S1.** Correlation of STXBP5-AS1 expression with clinicopathological features in 60 pancreatic cancer patients.

## Data Availability

Not applicable.

## Abbreviations

LncRNA: Long non-coding RNA
PC: Pancreatic cancer
GR: Gemcitabine-resistant
RIP: RNA-immunoprecipitation
ChIP: Chromatin immunoprecipitation
