# Supplementary material for: LncRNA STXBP5-AS1 suppresses stem cell-like properties of pancreatic cancer by epigenetically inhibiting neighboring androglobin gene expression
Source: Clin Epigenetics. 2020 Nov 7;12:168. doi: 10.1186/s13148-020-00961-y (PMC7648265; doi:10.1186/s13148-020-00961-y)
Supplement: Supplementary file 1 — Additional file 1: Table S1. Correlation of STXBP5-AS1 expression with clinicopathological features in 60 pancreatic cancer patients. [file 13148_2020_961_MOESM1_ESM.docx]

**Supplementary materials**

**Table S1.** Correlation of STXBP5-AS1 expression with clinicopathological features in 60 pancreatic cancer patients

| Expression of miR-876-3p | | | |
| --- | --- | --- | --- |
| Variables Low (%) High (%) P value | | | |
| Age |  |  | 0.9445 |
| <60 | 14(53.85%) | 12(46.15%) |  |
| ≥60 | 18(52.94%) | 16(47.06%) |  |
| Gender |  |  | 0.5921 |
| Male | 18(47.37%) | 20(52.63%) |  |
| female | 12(54.55%) | 10(45.45%) |  |
| TNM staging |  |  | 0.0402 |
| Ⅰ-Ⅱ | 10(37.04%) | 17(62.96%) |  |
| Ⅲ-Ⅳ | 21(63.64%) | 12(36.36%) |  |
| Tumor size (cm) |  |  | 0.1965 |
| <5 | 13(41.94%) | 18(58.06%) |  |
| ≥5 | 17(58.62%) | 12(41.38%) |  |
| Histological |  |  | 0.0207 |
| Well | 11(34.38%) | 21(65.63%) |  |
| Moderate and poor | 18(64.29%) | 10(35.71%) |  |
| Lymph node status |  |  | 0.0167 |
| Positive | 20(71.43%) | 8(28.57%) |  |
| Negative | 13(40.63%) | 19(59.38%) |  |
